# Sailuotong Prevents Hydrogen Peroxide (H_2_O_2_)-Induced Injury in EA.hy926 Cells

**DOI:** 10.3390/ijms18010095

**Published:** 2017-01-05

**Authors:** Sai Wang Seto, Dennis Chang, Wai Man Ko, Xian Zhou, Hosen Kiat, Alan Bensoussan, Simon M. Y. Lee, Maggie P. M. Hoi, Genevieve Z. Steiner, Jianxun Liu

**Affiliations:** 1 National Institute of Complementary Medicine (NICM), Western Sydney University, Penrith, NSW 2571, Australia; d.chang@westernsydney.edu.au (D.C.); i.ko@westernsydney.edu.au (W.M.K.); P.Zhou@westernsydney.edu.au (X.Z.); A.Bensoussan@westernsydney.edu.au (A.B.); G.Steiner@westernsydney.edu.au (G.Z.S.); Jianxun.Liu@westernsydney.edu.au (J.L.); 2 Faculty of Medicine, University of New South Wales, NSW 2052, Australia; hosen.kiat@chi.org.au; 3 School of Medicine, Western Sydney University, Locked Bag 1797, Penrith, NSW 2751, Australia; 4 Faculty of Medicine and Health Sciences, Macquarie University, NSW 2109, Australia; 5 State Key Laboratory Research in Chinese Medicine and Institute of Chinese Medical Sciences, University of Macau, Macao, China; simonlee@umac.mo (S.M.Y.L.); maghoi@umac.mo (M.P.M.H.); 6 Xiyuan Hospital, China Academy of Chinese Medical Sciences, Beijing 100091, China

**Keywords:** reactive oxygen species, Sailuotong (SLT), herbal medicine, apoptosis, endothelial dysfunction, vascular dementia

## Abstract

Sailuotong (SLT) is a standardised three-herb formulation consisting of *Panax ginseng*, *Ginkgo biloba*, and *Crocus sativus* designed for the management of vascular dementia. While the latest clinical trials have demonstrated beneficial effects of SLT in vascular dementia, the underlying cellular mechanisms have not been fully explored. The aim of this study was to assess the ability and mechanisms of SLT to act against hydrogen peroxide (H_2_O_2_)-induced oxidative damage in cultured human vascular endothelial cells (EAhy926). SLT (1–50 µg/mL) significantly suppressed the H_2_O_2_-induced cell death and abolished the H_2_O_2_-induced reactive oxygen species (ROS) generation in a concentration-dependent manner. Similarly, H_2_O_2_ (0.5 mM; 24 h) caused a ~2-fold increase in lactate dehydrogenase (LDH) release from the EA.hy926 cells which were significantly suppressed by SLT (1–50 µg/mL) in a concentration-dependent manner. Incubation of SLT (50 µg/mL) increased superoxide dismutase (SOD) activity and suppressed the H_2_O_2_-enhanced Bax/Bcl-2 ratio and cleaved caspase-3 expression. In conclusion, our results suggest that SLT protects EA.hy916 cells against H_2_O_2_-mediated injury via direct reduction of intracellular ROS generation and an increase in SOD activity. These protective effects are closely associated with the inhibition of the apoptotic death cascade via the suppression of caspase-3 activation and reduction of Bax/Bcl-2 ratio, thereby indicating a potential mechanism of action for the clinical effects observed.

## 1. Introduction

Cerebrovascular disease (CVD), such as stroke and vascular dementia, is a leading cause of morbidity and mortality, imposing a huge financial burden on the health care system worldwide. Progression of CVD is mediated via a numbers of factors, such as aging, hypertension, inflammation, and atherosclerosis, over a prolonged period [1,2]. It is now well established that many of these factors are closely associated with a chronic increase of oxidative stress, which can lead to vascular endothelial damage [3,4]. Oxidative stress is caused by excessive generation of reactive oxygen species (ROS), including superoxide anions and hydroxyl radicals. Hydrogen peroxide (H_2_O_2_) is one of the major ROS and it has been shown to be generated during ischemia-reperfusion injury and in animal models of chronic brain disorders [5,6]. In vitro studies have demonstrated that H_2_O_2_ can induce oxidative stress in endothelial cells, causing cellular dysfunction and apoptosis [7].

Interestingly, recent studies have demonstrated a close linkage between neurological dysfunction and vascular disease, highlighting the crucial role of cell–cell signalling between neurons, astrocytes, and endothelial cells within the neurovascular unit (NVU) for normal brain functions [8,9]. It has been suggested that the cerebral endothelium and microvessels system are not just “empty pipes” for blood circulation, but instead act as an intricate paracrine organ that supports neuronal functions and homeostasis [10]. Numerous studies have suggested that endothelial cells play a fundamental role in various physiological and pathological processes. For example, Sorriento et al. demonstrated that endothelial cells are able to synthesise and release catecholamine in response to ischemia, highlighting their vital roles in the control of vascular homeostasis [11]. A recent report also suggested that microRNA contributes significantly to various therapeutic approaches to preserve endothelial integrity and vascular health [12], such as vascularization, a process that is closely associated with ROS-dependent signalling [13,14]. Moreover, salidroside, a glycoside from *Rhodiola rosea*, has been shown to reduce H_2_O_2_-induced ROS generation via the upregulation of miR-103 [15]. Hence, interventions that can suppress ROS-induced endothelial cell damage would be beneficial for CVDs as well as neurological disorders.

Herbal medicine has been widely used in CVD management in many Asian countries for centuries. A large body of evidence has shown that many herbs have remarkable anti-oxidative properties [16]. Sailuotong (SLT) is a standardised three-herb formula combining specific dosages of *Panax ginseng* (ginseng), *Ginkgo biloba* (ginkgo), and *Crocus sativus* (saffron) for the management of vascular dementia (VaD) [17,18]. The chemical profile and optimal ratio of the three herbal extracts have been determined and studied in detail previously [19]. In a chronic cerebral hypoperfusion model induced by bilateral common carotid artery ligation in rats, an eight week treatment of SLT (ig) significantly shortened the persistent time for finding the platform in a Morris Water Maze task. This beneficial effect was found to be associated with an increased acetylcholine level and superoxide dismutase (SOD) activity in the brain [20]. SLT (8.25, 16.5, and 33 mg/kg over 24 h) has been shown to significantly decrease the areas of focal cerebral ischemia/reperfusion injury by increasing cerebral blood flow in anesthetized dogs [21]. Moreover, SLT treatment (16 mg/kg and 8 mg/kg for seven days) also significantly decreased the platelet aggregation rate and whole blood viscosity in rabbits [21].

Cerebral and vascular protective effects of the individual components of SLT have been demonstrated previously. For instance, crocin, the principal active component of *Crocus sativus*, has been shown to act against cerebral ischemia/reperfusion (I/R) damage via an increase of SOD and glutathione peroxidase (GSH-px) activities and a reduction of metalloproteinase-9 (MMP-9) expression in a global cerebral ischemia mouse model [22]. Similarly, ginsenoside Rb1, a natural steroid glycoside from *Panax ginseng*, has been shown to reduce brain damage caused by middle cerebral artery occlusion (MCAO) via reduction of cerebral oxidative stress in aged mice [23]. EGb761, a standardised extract of *Ginkgo biloba*, has been shown to protect bovine vascular endothelial cells against oxidative stress-induced damage via reduced intracellular ROS generation and the regulation of apoptosis-related genes expression, including Bax and Bcl-2 [24,25].

It is worth noting that, despite a recent clinical study demonstrating that SLT improved cognitive function in people with VaD [18], and numerous pre-clinical studies demonstrating the cerebrovascular protective effects of its individual components [23,26,27], the cellular mechanisms of SLT in protecting endothelial cells from oxidative stress have not been previously studied. Thus, the aims of this study were to investigate the effects of SLT on H_2_O_2_-induced endothelial cell injury using cultured human umbilical vein cell line, EA.hy926, and to explore the mechanisms of action underlying these effects.

## 2. Results

### 2.1. Effects of SLT on the Viability of EA.hy926 Cells Injured by H_2_O_2_

Cytotoxicity of SLT (0.1–200 µg/mL; *n* = 3) on EA.hy926 cells was examined using MTT (3-(4,5-di-methylthiazol-2-yl) 2,5-diphenyltetrazolium bromide) assay. SLT did not show any significant cytotoxic effects up to 50 µg/mL [28]. Therefore, all the subsequent experiments were conducted at doses no higher than 50 µg/mL SLT.

**Figure 1:**
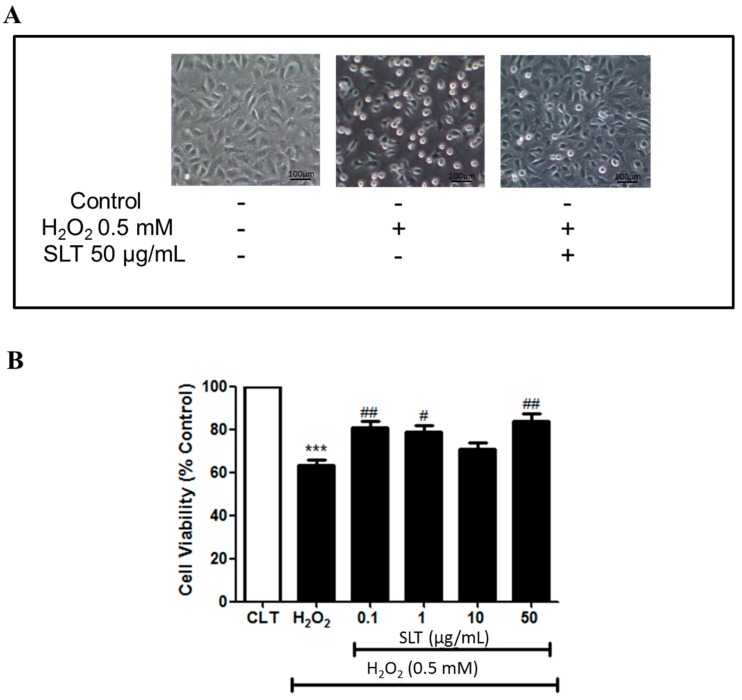
(**A**) Representative images of the effect of Sailuotong (SLT) (50 µg/mL) on EA.hy926 cell morphology injured by H_2_O_2_ observed under an inverted/phase contract microscope; (**B**) Effect of Sailuotong (SLT) (0.1–50 µg/mL) on EA.hy926 cells viability injured by H_2_O_2_ (*n* = 3) measured by MTT assay. Data are presented as means ± S.D. *** *p* < 0.001 vs. control group; # *p* < 0.05 vs. H_2_O_2_ group; ## *p* < 0.01 vs. H_2_O_2_ group.

To evaluate whether SLT could act against H_2_O_2_-induced cell damage, cells were pre-incubated with SLT for 60 min, then challenged by H_2_O_2_ (0.5 mM) for 24 h; cell viability was measured by MTT assay. EA.hy926 cell viability was markedly reduced by H_2_O_2_ (0.5 mM; 24 h) (*p* < 0.001, *n* = 3). Pre-incubation of SLT (0.1–50 µg/mL) protected cells from H_2_O_2_-induced cell damage (*p* < 0.01 at 50 µg/mL; *n* = 3) (Figure 1A,B). These results indicate that SLT could protect EA.hy926 cells from ROS-related cellular damage.

### 2.2. Effects of SLT on LDH Leakage and SOD Activity in H_2_O_2_ Treated EA.hy926 Cells

**Figure 2:**
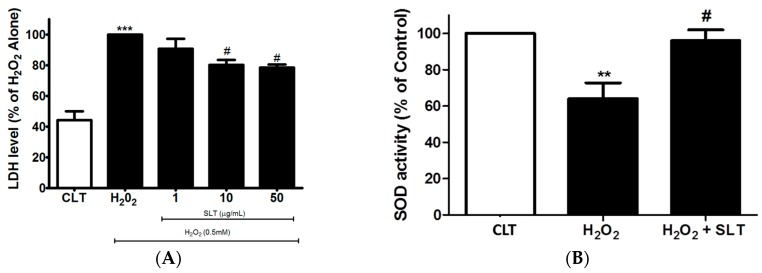
(**A**) Effects of SLT (1–50 µg/mL) on H_2_O_2_-induced lactate dehydrogenase (LDH) leakage in EA.hy926 cells (*n* = 3). Data are presented as means ± S.D. *** *p* < 0.001 vs. control (CLT) group; # *p* < 0.05 vs. H_2_O_2_ group; (**B**) Effects of SLT (50 µg/mL) on H_2_O_2_-inhibited superoxide dismutase (SOD) activity in EA.hy926 cells (*n* = 3). Data are presented as means ± S.D. ** *p* < 0.01 vs. control (CLT) group; # *p* < 0.05 vs. H_2_O_2_ group.

Lactate dehydrogenase (LDH) is one of the major representative indicators of cell injury. Therefore, the protective effect of SLT on H_2_O_2_-treated EA.hy926 cells was confirmed using LDH assay. As shown in Figure 2A, H_2_O_2_ (0.5 mM; 24 h) markedly increased LDH leakage from the EA.hy926 cells (*p* < 0.05, *n* = 3), while SLT reduced this H_2_O_2_-mediated LDH leakage in a concentration-dependent manner (*p* < 0.05 at 50 µg/mL compared to H_2_O_2_ alone; *n* = 3).

To further examine the protective effects of SLT, we measured SOD activity in H_2_O_2_-treated EA.hy926 cells. SOD activity was significantly reduced by H_2_O_2_ (*p* < 0.05 compared to control, *n* = 3). This significant reduction of SOD activity was partly reversed by SLT at 50 µg/mL (Figure 2B).

### 2.3. Effect of SLT on the Intracellular ROS Generation in H_2_O_2_ Treated EA.hy926 Cells

**Figure 3:**
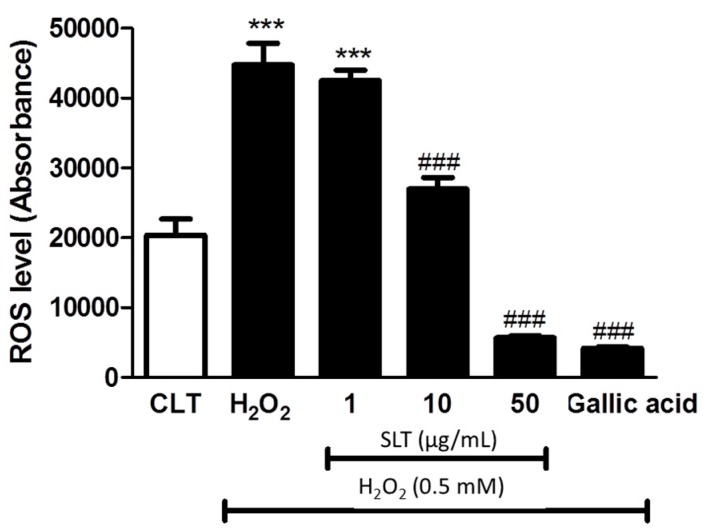
Effects of SLT (1–50 µg/mL) on H_2_O_2_-induced intracellular reactive oxygen species (ROS) generation in EA.hy926 cells. Gallic acid (10 µg/mL), a known potent anti-oxidant, was used as a positive control (*n* = 3). Data are presented as means ± S.D. *** *p* < 0.05 vs. control group; ### *p* < 0.001 vs. H_2_O_2_ group.

In order to elucidate whether the protective effect of SLT is mediated by a reduction of intracellular oxidative stress, intracellular ROS generation was determined by 2′,7′-Dichlorofluorescin diacetate (DCFH-DA), ROS specific dye. As shown in Figure 3, H_2_O_2_ markedly increased (~2-fold) intracellular ROS generation in EA.hy926 cells (*p* < 0.001 compared to control, *n* = 3) and SLT (1–50 µg/mL) suppressed this H_2_O_2_-induced ROS generation in a concentration-dependent manner (*p* < 0.001 at 50 µg/mL compared to H_2_O_2_ alone; *n* = 3). Interestingly, the effect of SLT at 50 µg/mL in suppressing H_2_O_2_-induced ROS generation is comparable to gallic acid (10 µg/mL), a known potent anti-oxidant [29].

### 2.4. Effect of SLT on Protein Expression Level of Bax, Bcl-2, and Cleaved Caspase-3 in H_2_O_2_ Treated EA.hy926 Cells

**Figure 4:**
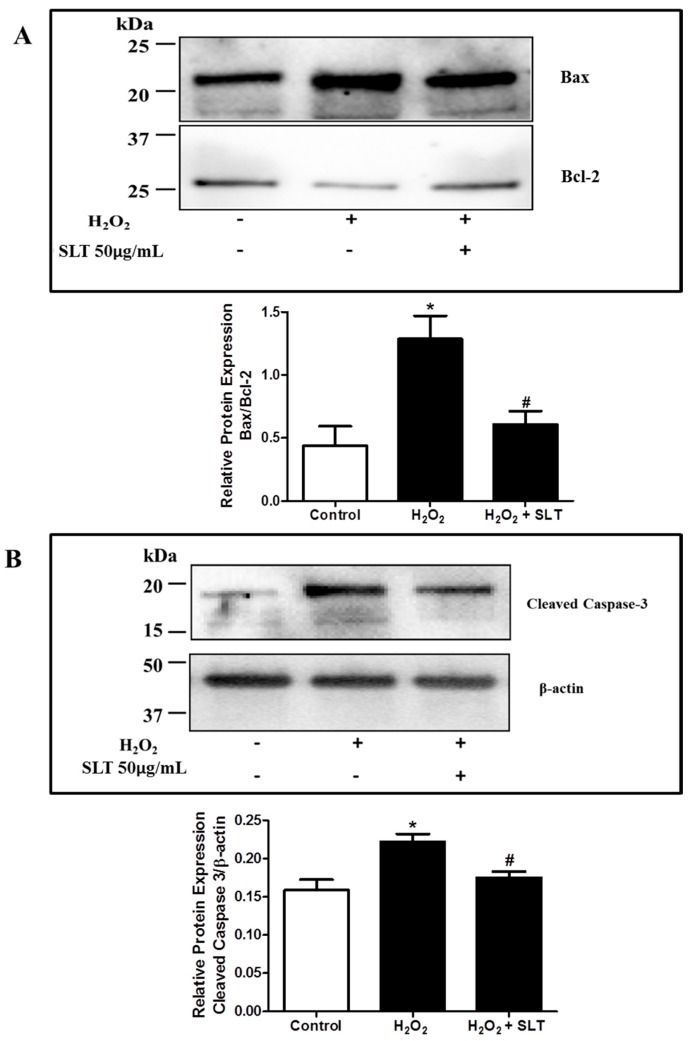
(**A**) Effects of SLT (50 µg/mL) on H_2_O_2_-upregulated Bax/Bcl-2 ratio in EA.hy926 cells (*n* = 3). Data are presented as means ± S.D. * *p* < 0.05 vs. control group; # *p* < 0.05 vs. H_2_O_2_ group. Images are representative of three independent experiments. The present or absent of H_2_O_2_ and SLT in the culture is indicated by + or − respectively; (**B**) Effects of SLT (50 µg/mL) on H_2_O_2_-upregulated cleaved caspase-3 expression in EA.hy926 cells (*n* = 3). Result was expressed as expression of cleaved caspase-3 protein relative to β-actin. Data are presented as means ± S.D. * *p* < 0.05 vs. control group; # *p* < 0.05 vs. H_2_O_2_ group. Images are representative of three independent experiments. The present or absent of H_2_O_2_ and SLT in the culture is indicated by + or − respectively.

The protein expressions of Bax (pro-apoptotic factor) and Bcl-2 (anti-apoptotic factor) in H_2_O_2_ treated EA.hy926 cells were evaluated using Western blotting with the result expressed as Bax/Bcl-2 ratio. As shown in Figure 4A, H_2_O_2_ treatment markedly increased the Bax/Bcl-2 ratio (*p* < 0.05 compared to control, *n* = 3), while SLT at 50 µg/mL reduced this H_2_O_2_-mediated effect significantly (*p* < 0.05 compared to H_2_O_2_ alone; *n* = 3). The effect of SLT on cleaved caspase-3 protein expression was also determined using Western blotting. It was found that H_2_O_2_ caused a significant increase of cleaved capsase-3 expression (*n* = 3) in EA.hy926 cells, and this effect was completely reversed by SLT at 50 µg/mL (*n* = 3) (Figure 4B).

## 3. Discussion

It is well established that endothelial dysfunction caused by elevated cerebrovascular oxidative stress is one of the major mechanisms of CVDs [3,6,30]. Therefore, interventions that can protect endothelial cells from ROS-induced damage would be beneficial for CVDs. In the present study, we demonstrated that SLT could protect EA.hy926 cells from oxidative stress and cell damage caused by H_2_O_2_. Furthermore, our results suggest that this effect is possibly mediated by a reduction of the Bax/Bcl-2 ratio and an increase of SOD activity in the EA.hy926 cells.

ROS, such as superoxide anions and hydroxyl radicals, are generated during normal cellular metabolism [31]. Under normal physiological conditions, vascular endothelial cells are in continuous contact with steady-state levels of oxidative metabolites. This constant level of ROS is tightly regulated by a number of anti-oxidative enzymes, such as SOD and glutathione peroxidase [32,33]. However, when this anti-oxidative mechanism is disrupted, excessive ROS will be generated, leading to endothelium dysfunction, which contributes to the development and progression of a number of cerebral and vascular diseases, such as atherosclerosis, stroke, and hypertension [6,34,35]. H_2_O_2_ is one of the ROS that have been shown to play a major role in vascular and endothelial dysfunction [3,10,36]. Numerous in vitro studies have demonstrated that high levels of H_2_O_2_ can cause significant injury and reduce endothelial cell viability [37,38,39]. In line with this, our results showed that H_2_O_2_ (0.5 mM, 24 h) significantly reduced viability and increased LDH leakage in the EA.hy926 cells. Interestingly, pre-incubation of the cells with SLT one hour prior to the addition of H_2_O_2_ significantly suppressed the H_2_O_2_-mediated cell death and LDH leakage, demonstrating the anti-apoptotic property of SLT.

A recent clinical study demonstrated that SLT can improve cognitive function and memory in people with vascular dementia [18]. Animal studies have suggested these clinically beneficial effects are possibly associated with increases in cerebral blood flow and reductions in platelet aggregation rate and whole blood viscosity [21]. However, the underlying cellular mechanisms of SLT in protecting endothelial cell from ROS-mediated injury had not been explored previously. H_2_O_2_ has been shown to induce oxidative stress via an increase in the generation of intracellular ROS in endothelial cells [40]. Our results showed that SLT suppressed the H_2_O_2_-induced intracellular ROS generation in a concentration-dependent manner. More importantly, SLT at 50 µg/mL produced a similar effect to our positive control, gallic acid (a known potent anti-oxidant) [29]. In addition, SOD is a major enzyme that protects against oxidative stress damage in endothelial cells [33,41]. In the present study, SLT at 50 µg/mL partly reversed the H_2_O_2_-suppressed SOD activity in the EA.hy926 cells. These results highlight the potent anti-oxidant properties of SLT through reduction in intracellular ROS generation and modulation of anti-oxidative enzyme activity.

A number of apoptosis-related proteins, including Bax, Bcl-2, and caspase-3, are required for cellular survival regulation [42]. In this study, we examined if SLT can suppress the apoptotic signalling pathway transduction triggered by H_2_O_2_. Our results showed that H_2_O_2_ increased the Bax/Bcl-2 ratio; this H_2_O_2_-induced effect was inhibited by pre-treatment of SLT. It has been demonstrated that intracellular ROS can increase cytosolic caspases activity via activation of Bax and dissociation of cytochrome C from the inner mitochondrial membrane [43]. Several studies have suggested that Bax/Bcl-2 plays a role in determining cell apoptosis process [44,45]. H_2_O_2_ has been shown to downregulate Bcl-2 and upregulate Bax (i.e., increased Bax/Bcl-2 ratio), leading to caspase-3 cleavage, and eventually apoptosis [46]. Caspases-3 is one of the most important enzymes responsible for the cleaving of many cellular substrates during apoptosis [47]. In line with this, we demonstrated that the anti-apoptotic effect of SLT appears to be associated with the inhibition of the apoptotic death cascade via suppression of caspase-3 activation and a reduction in the Bax/Bcl-2 ratio in EA.hy926 cells.

Endothelial dysfunctions are closely associated with vascular dementia and other neurological disorders [9,30]. For instance, changes in brain vascular endothelial cell morphology can reduce the blood-brain barrier permeability, leading to cognitive decline and dementia [48]. Moreover, an increase in ROS generation and oxidative stress in vasculature has been suggested as one of the central pathologies of both vascular dementia and Alzheimer’s disease [49]. Given that current therapies for these diseases are limited [50,51], the development of new therapies/interventions is urgently needed. In this regard, SLT has recently been demonstrated to improve cognitive function and memory in people with vascular dementia. Despite the relatively small sample size in this study, the findings have highlighted the therapeutic potential of SLT in improving cognitive function in people with dementia [18]. It is important to point out that, although some preclinical pharmacokinetic, toxicity, and pharmacodynamics studies of SLT and its individual components have been conducted in several animal models of cerebral disorders [20,21,22,27,52], the cellular and molecular effects of SLT in endothelial cells have not been fully explored. The results of this study demonstrate the effects and underlying signalling mechanisms of SLT against oxidative stress in EA.hy926 cells, providing new insights and molecular evidence to previous in vivo and clinical observations.

The present study has several limitations. Multiple sources of ROS have been suggested to contribute to endothelial cell damage [53,54]. For example, Xie et al. demonstrated the significant role of mitochondrial-derived ROS in age-related cardiovascular diseases [55]. Our current experiments have only examined the global cellular ROS generation in response to exogenous H_2_O_2_; more detailed studies, such as direct measurement of mitochondrial ROS, are required to clarify the cellular target and anti-oxidative property of SLT in endothelial cell. Although our results show that the anti-apoptotic property of SLT was associated with reduction in caspase-3 activation and Bax/Bcl-2 ratio, the effects of SLT on other cellular signalling pathways should also be explored using additional experimental models (e.g., heavy metal-induced [56] and hypoxia-induced [57] oxidative stress models). Additionally, contributions of individual components of SLT to the observed effects were not determined.

## 4. Materials and Methods

### 4.1. Reagents and Antibodies

SLT extracts were provided in-kind by Shineway Pharmaceutical Group (Shijiazhuang, China). Hydrogen peroxide (H_2_O_2_), dimethylsulfoxide (DMSO), and trypan blue were purchased from Sigma-Aldrich (St. Louis, MO, USA). Dulbecco’s Modified Eagle’s Medium Ham’s F-12 (DMEM/F12) (1:1 Mix) with L-Glutamine was purchased from Lonza (Basel, Switzerland). Fetal bovine serum (FBS) and penicillin and streptomycin (PS) were purchased from Gibco Life Technologies (Waltham, MA, USA). 3-(4,5-di-methylthiazol-2-yl) 2,5-diphenyltetrazolium bromide (MTT) was purchased from Astral Scientific (Caringbah, Australia). The superoxide dismutase (SOD) activity assay kit was purchased from Cayman Chemical (Ann Arbor, MI, USA). CytoTox non-radioactive cytotoxicity assay was obtained from Promega Corporation (Madison, WI, USA). The cellular ROS/Superoxide detection assay kit was purchased from Abcam (Cambridge, UK). Anti-Bax, anti-Bcl-2, anti-cleaved caspase-3, and anti-beta actin antibodies were obtained from Santa Cruz Biotechnology (Dallas, TX, USA). All other reagents and chemicals were of chemical analytical grade.

### 4.2. EA.hy 926 Cell Culture

The permanent human endothelial cell line EA.hy926 was originally derived from a human umbilical vein obtained from ATCC (Manassas, VA, USA). In this study, cells were grown in DMEM/F12 (1:1 Mix) supplemented with 10% fetal bovine serum (FBS), 1% l-glutamine, and 1% penicillin-streptomycin in a humidified atmosphere of 5% CO_2_ at 37 °C. During cell culture, the medium was changed every three days until the cells reached 90% confluence. To assess the effects of SLT on EA.hy926 cells, the cells were treated with increasing concentrations of SLT (0.1, 1, 10, 50 µg/mL) for 1 h followed by H_2_O_2_ (0.5 mM) or vehicle for 24 h unless stated otherwise.

### 4.3. Measurement of Cell Viability

Cell viability was determined using MTT assay. In brief, cells were seeded in 96-well plates at a density of 1.0 × 10^5^ cells/well and allowed to attach for 24 h. After incubation with the above-mentioned treatments, the culture supernatant was removed, then the cells were incubated with MTT (5 mg/mL) in DMEM/F12 medium at 37 °C for 4 h. After MMT incubation, the culture medium with dye was replaced with 150 µL DMSO and was agitated in a plate shaker for 5 min. Next, the optical density (O.D.) of each well was measured at 560 nm using a Microplate Reader (BMG Labtech, Ortenberg, Germany). Cell viability was expressed as a percentage relative to control.

### 4.4. Measurement of Intracellular ROS Level

Intracellular ROS level was evaluated using the cellular ROS/superoxide detection assay kit (Abcam, Cambridge, UK) according to the manufacturer’s instructions. In brief, cells were seeded in 96-well plates at a density of 1.0 × 10^5^ cells/well and allowed to attach for 24 h. After incubation with the above-mentioned treatments, the culture supernatant was removed and the cells were washed with 100 µL/well of 1× assay buffer. The ROS specific stain, DCFH-DA, was added to the cells and allowed to incubate in the dark for 60 min. After the incubation, intracellular ROS level was determined using a fluorescence microplate reader (*Ex* = 488 nm, *Em* = 520 nm) (BMG Labtech, Ortenberg, Germany).

### 4.5. Measurement of Lactate Dehydrogenase (LDH) Leakage

Lactate dehydrogenase (LDH) release was evaluated using the non-radioactive assay kit (Promega Corporation, Madison, WI, USA) according to the manufacturer’s instructions. Briefly, cells were seeded in 96-well plates at a density of 1.0 × 10^5^ cells/well and allowed to attach for 24 h. After incubation with the above-mentioned treatments, 50 µL of supernatant per well was transferred to a 96-well flat clear bottom plate. An equal amount of CytoTox reagent was added to each well and allowed to incubate for 30 min at room temperature. A stop solution was added to terminate the reaction at the end of the incubation period. LDH level was measured at 490 nm using a microplate reader (BMG Labtech, Ortenberg, Germany).

### 4.6. Measurement of Intracellular SOD Activity 

Cells were seeded in six-well plates at a density of 1.0 × 10^6^ cells/well and allowed to attach for 24 h. The cells were treated with vehicle, H_2_O_2_ (0.5 mM; 24 h) alone or H_2_O_2_ (0.5 mM; 24 h) with SLT (50 µg/mL; 1 h prior addition of H_2_O_2_). After the treatment, the cells were lysed by Freeze Thaw method three times. The activity of SOD was determined using a commercially available kit, according to the manufacturer’s instructions.

### 4.7. Western Blotting

Cells were seeded in six-well plates at a density of 1.0 × 10^6^ cells/well and allowed to attach for 24 h. The cells were treated with vehicle, H_2_O_2_ (0.5 mM; 24 h) alone or H_2_O_2_ (0.5 mM; 24 h) with SLT (50 µg/mL; 1 h prior addition of H_2_O_2_). After the treatment, the cells were homogenized and lysed in a Radioimmunoprecipitation assay RIPA buffer (Thermo Scientific, Waltham, MA, USA) in the presence of protease inhibitors (Roche Applied Science, Penzberg, Germany) to obtain protein extracts. Protein concentrations were determined using the bovine serum albumin (BSA) protein assay kit (Pierce, Waltham, MA, USA). Samples (25 µg of protein per lane) were loaded onto a mini-PROTEAN TGXTM precast electrophoresis gel (BioRad, Hercules, CA, USA). After electrophoresis (110 V, 90 min), the separated proteins were transferred to polyvinylidene difluoride (PVDF) membranes using iBlot 2 gel transfer system (Thermofisher, Waltham, MA, USA). Non-specific sites were blocked with 5% non-fat dry milk in Phosphate Buffered Saline Tween-20 (PBSt) for 60 min, and the blots were then incubated with anti-Bax, 1:1000 (Santa Cruz), anti-Bcl-2, 1:1000 (Santa Cruz), anti-cleaved caspase-3, 1:1000 (Santa Cruz, Dallas, TX, USA), and anti-beta actin, 1:10,000 (Santa Cruz) in PBSt overnight at 4 °C. Anti-rabbit horseradish peroxidase (HRP) conjugated immunoglobulin G (IgG), 1:1000 (DakoCytomation, Glostrup, Denmark) in PBSt (60 min, room temperature) was used to detect the binding of its correspondent antibody. β-actin was used to verify equal loading of protein in each lane. The protein expression was detected with Western Lightning Chemiluminescence Reagent Plus (PerkinElmer Life Sciences, Waltham, MA, USA) and quantified by Quantity One (version 4.6.7) software (BioRad).

### 4.8. Statistical Analysis

Data were presented as mean ± SEM of n experiments. Statistical comparisons were performed using t-test or one-way analysis of variance (ANOVA), where appropriate. Differences were considered to be statistically significant at *p* < 0.05. All statistical analyses were performed using GraphPad Prism 5 software (GraphPad Software, Inc., La Jolla, CA, USA).

## 5. Conclusions

In conclusion, our results revealed that SLT can inhibit H_2_O_2_-induced endothelial cell injury via the direct reduction of intracellular ROS generation and increase of SOD activity. These protective effects are closely associated with the inhibition of the apoptotic death cascade through suppression of caspase-3 activation and reduction of Bax/Bcl-2 ratio. Our data suggest that SLT possesses potent anti-oxidative and anti-apoptotic activities, which at least partially contribute to its cognitive enhancing effects observed in the clinical study. Further studies are required to investigate the effects and mechanisms of SLT between different cell types within the neurovascular unit and the interaction/synergistic effects between individual components of SLT
